# Impact of whole-body vibration on anthropometric measurements and body composition in overweight and obese university students: a randomized controlled trial

**DOI:** 10.3389/fmed.2026.1814883

**Published:** 2026-06-11

**Authors:** Rokaia A. Toson, Khloud Al-Ruwailiy, Raghad Al-Sarhany, Zeinab A. Ali, Hadeel Alsirhani, Nabeela Kashif, Rabab A. Mohamed, Amany E. Abd-Eltawab, Samaher Alowaydhah, Engi E. Sarhan, Mustafa Shukry, Eman Mohammed Elnashar, Hanan Hassan Alshehri, Rasha Hamed Al-Serwi, Mohamed Mahmoud Abdelfattah Abdelrahman, Nesma M. Allam

**Affiliations:** 1 Department of Physical Therapy and Health Rehabilitation, College of Applied Medical Sciences, Jouf University, Sakakah, Saudi Arabia; 2 Department of Physical Therapy for Surgery, Faculty of Physical Therapy, Cairo University, Giza, Egypt; 3 Department of Basic Science, Faculty of Physical Therapy, Cairo University, Giza, Egypt; 4 Department of Physical Therapy, College of Applied Medical Sciences, Qassim University, Buraidah, Saudi Arabia; 5 Department of Biomechanics, Faculty of Physical Therapy, Cairo University, Giza, Egypt; 6 Department of Physical Therapy for Neurology and its Surgery, Faculty of Physical Therapy, Kafrelsheikh University, Kafr El-Shaikh, Egypt; 7 Department of Biomedical Sciences, College of Veterinary Medicine, King Faisal University, Al-Ahsa, Saudi Arabia; 8 Department of Anatomy, College of Medicine, King Khalid University, Abha, Saudi Arabia; 9 Endocrinology and Diabetes Section, Department of Internal Medicine, College of Medicine, King Khalid University, Abha, Saudi Arabia; 10 Department of Basic Dental Sciences, College of Dentistry, Princess Nourah bint Abdulrahman University, Riyadh, Saudi Arabia; 11 Department of Anaesthesia and Critical Care, King Abdulaziz University Hospital, King Abdulaziz University, Jeddah, Saudi Arabia; 12 Department of Anaesthesia and Surgical Intensive Care, Faculty of Medicine, Mansoura University, Mansoura, Egypt

**Keywords:** anthropometric parameters, body composition, obesity, overweight, whole-body vibration

## Abstract

**Background:**

The prevalence of overweight and obesity among university students is a significant public health concern. Consequently, identifying safe exercise modalities for this population is a priority.

**Objectives:**

This study aimed to assess the efficacy of whole-body vibration (WBV) in university students classified as overweight or obese, focusing on anthropometric measurements and body composition.

**Materials and methods:**

A randomized controlled study was conducted with a sample of 30 university students (both male and female) aged 18–25 years. Participants were allocated into two groups: the intervention group (WBV group), which received whole-body vibration (WBV) therapy together with selected exercises, and the control group (Sham WBV group), which received sham WBV alongside the same exercises, administered thrice per week over a six-week period. The primary outcome measures included body mass index (BMI), assessed using a stadiometer and digital scale, and waist circumference (WC), measured using a tape measure. Secondary outcomes were evaluated using bioelectrical impedance analysis (BIA) via a smart scale to determine the whole-body fat percentage (BF%), visceral fat, and lean mass (kg).

**Results:**

Participants in the WBV group demonstrated a statistically significant decrease in both BMI and WC compared to those in the sham group (*p* = 0.028 and *p* = 0.003, respectively). In contrast, no significant between-group differences were identified for body fat percentage, visceral fat, or lean body mass (*p* = 0.884, 0.29, and 0.199, respectively). These findings suggest that WBV may preferentially affect central fat distribution rather than overall body composition.

**Conclusion:**

WBV positively impacted the anthropometric measurements of overweight and obese university students, as observed by improvements in BMI and WC.

**Clinical trial registration:**

https://clinicaltrials.gov/, identifier NCT06913231.

## Introduction

The significant global increase in the prevalence of overweight and obesity in recent decades is a major public health concern (1). These conditions have become increasingly prevalent across various age groups, particularly among the younger population (2). University students are significantly affected by overweight and obesity (3). Data from 22 countries, representing diverse socioeconomic backgrounds and geographic regions, indicate that approximately 22 percent of university students are classified as overweight or obese (4). These conditions can adversely affect an individual’s social, psychological, and physical health, elevating the risk of hyperglycemia (5), hypertension (6), cardiovascular diseases (7), osteoarthritis (8), several types of cancer (9), and mortality (10). Numerous studies have highlighted the importance of preventing obesity in young people. First, early age BMI serves as a predictor of obesity in adulthood (11). Second, the correlation between obesity and mortality is more pronounced at younger ages than at older ages (12). One notable outcome is the weight gain observed in first-year college students, commonly referred to as the “Freshman 15” (13). Research indicates that weight gain among first-year university students is significantly influenced by factors such as sex, ethnicity, dietary habits, and physical activity (14).

A decrease of 5–10% in body weight among individuals with obesity is associated with a decreased risk of cardiovascular disease (15). Consequently, the primary goal of treatment is weight reduction, which can be achieved through dietary modifications (16) and/or implementing exercise regimens (17). Exercise, including aerobic and resistance training, enhances physical strength, modifies body composition, and reduces body weight and cardiometabolic risk (18). However, many individuals with obesity tend to lead a sedentary lifestyle. They often exhibit reluctance to adhere to exercise routines because of factors such as musculoskeletal pain, low self-motivation, concurrent physical limitations (19), and reduced cardiorespiratory fitness (20).

Previous research involving animal models, specifically rats, has indicated that whole-body vibration (WBV) may inhibit adipogenesis when applied for a sufficient duration. WBV therapy has been suggested as an alternative to traditional exercise for reducing body fat and improving muscle mass and strength in obese individuals (21).

Whole-body vibration (WBV) represents a passive workout in which a mechanical stimulus, known as vibration, produces a force in the muscle through oscillatory motion (22, 23). The platform vibrates based on the product at various frequencies (1–60 Hz) and displacement amplitudes (1–10 mm) (24). Considering the localized or systemic physiological effects, WBV programs have been clinically validated across domains, ranging from muscle performance to general health (25). Numerous systematic reviews and review articles have examined the efficacy of WBV in patients with neuromuscular and musculoskeletal disorders. Among these, WBV may help women with postmenopausal osteoporosis increase their bone mineral density (26), enhance neuromuscular function (27), and improve motor function in neurological conditions (28). In addition, it can influence the body composition of healthy individuals who are overweight or obese (21).

Previous research on WBV has generally examined individual body composition indicators, such as BMI (29), visceral fat (30), or lean mass (31), separately. However, there is limited research evaluating multiple body composition measurements simultaneously within a single study, particularly among college students with overweight and obesity. Consequently, this randomized controlled trial aimed to examine the effects of a 6-week whole-body vibration training program on anthropometric measurements (BMI and waist circumference) and body composition measurements (BF%, visceral fat, and lean mass) in overweight and obese university students.

## Materials and methods

### Study design

A total of 38 students from Jouf University participated in this prospective randomized controlled trial, which was conducted at the Physical Therapy and Rehabilitation Department of Prince Mutaib bin Abdul Aziz General Hospital in Jouf, Saudi Arabia. Recruitment was conducted on the university campus through announcements and advertisements. Participation was voluntary, and interested students were invited to a screening session in which eligibility was assessed based on predefined inclusion and exclusion criteria. Of the 38 students initially evaluated, 30 met all the criteria and were randomly assigned to either the WBV or sham WBV group. The study was conducted between April and August 2025. This trial was registered with the Clinical Trials Registry (NCT06913231), and the study design and procedures received ethical approval from the Jouf University Ethics Committee (No. 7586).

### Subjects

Eligible participants for this study were male or female university students aged 18–25 years, with a Body Mass Index (BMI) ranging from 25 to 40 kg/m^2^. The exclusion criteria included a history of cardiorespiratory or neuromuscular disorders, engagement in strength training, competitive sports, or structured fitness programs, adherence to any diet or weight loss program, presence of a pacemaker, or pregnancy. The participants were instructed to maintain their usual physical activity and dietary habits throughout the trial.

### Sample size

**FIGURE 1:**
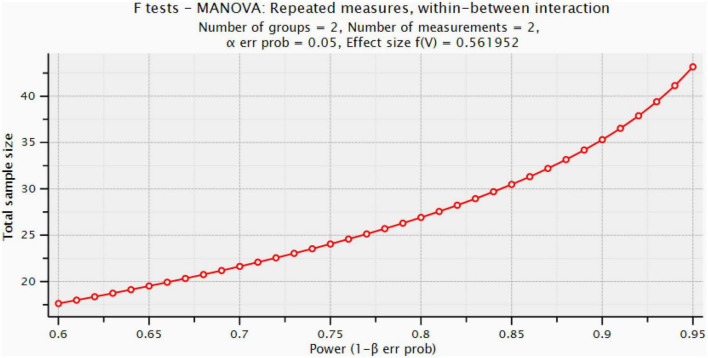
Plot of sample size estimation.

The G*Power software program (version 3.1.9.4, Dusseldorf, Germany) was used to perform a power analysis. Given two groups and two measurements [*F*-tests- MANOVA: Repeated measures-between interaction, number of measurements = 2, and number of dependents = 5] with a statistical power of 80% (1-β error probability), β = 0.20, α = 0.05 for type I error, and an effect size of *F* = 0.56. A total sample size of 27 was considered sufficient. To account for dropouts, the sample size was increased by 10% to 30 participants from the original predicted sample size of 27 participants. This effect size was derived from data obtained from a pilot study involving 10 participants (five in the study group and five in the control group) (Pillai *V* = 0.24) for all dependent variables as primary outcomes, which were not included in the final analysis (Figure 1).

### Randomization

All participants received comprehensive information regarding the study objectives, procedures, and significance. They were also informed of their right to withdraw voluntarily at any time and of the measures implemented to ensure the confidentiality of their data. Randomization was conducted using a computer-generated sequence created with IBM SPSS Statistics software (Version XX, IBM Corp., Armonk, NY, United States), with participants allocated in a 1:1 ratio. Of the 38 individuals assessed for eligibility, 30 who met the predefined inclusion criteria were randomly assigned to either the WBV training or sham WBV group. The randomization sequence was prepared by an independent researcher who was not involved in participant recruitment, assessments, or data analysis. Allocation concealment was ensured using sequentially numbered, sealed, opaque envelopes that were opened only after the completion of baseline screening.

### Blinding

**FIGURE 2:**
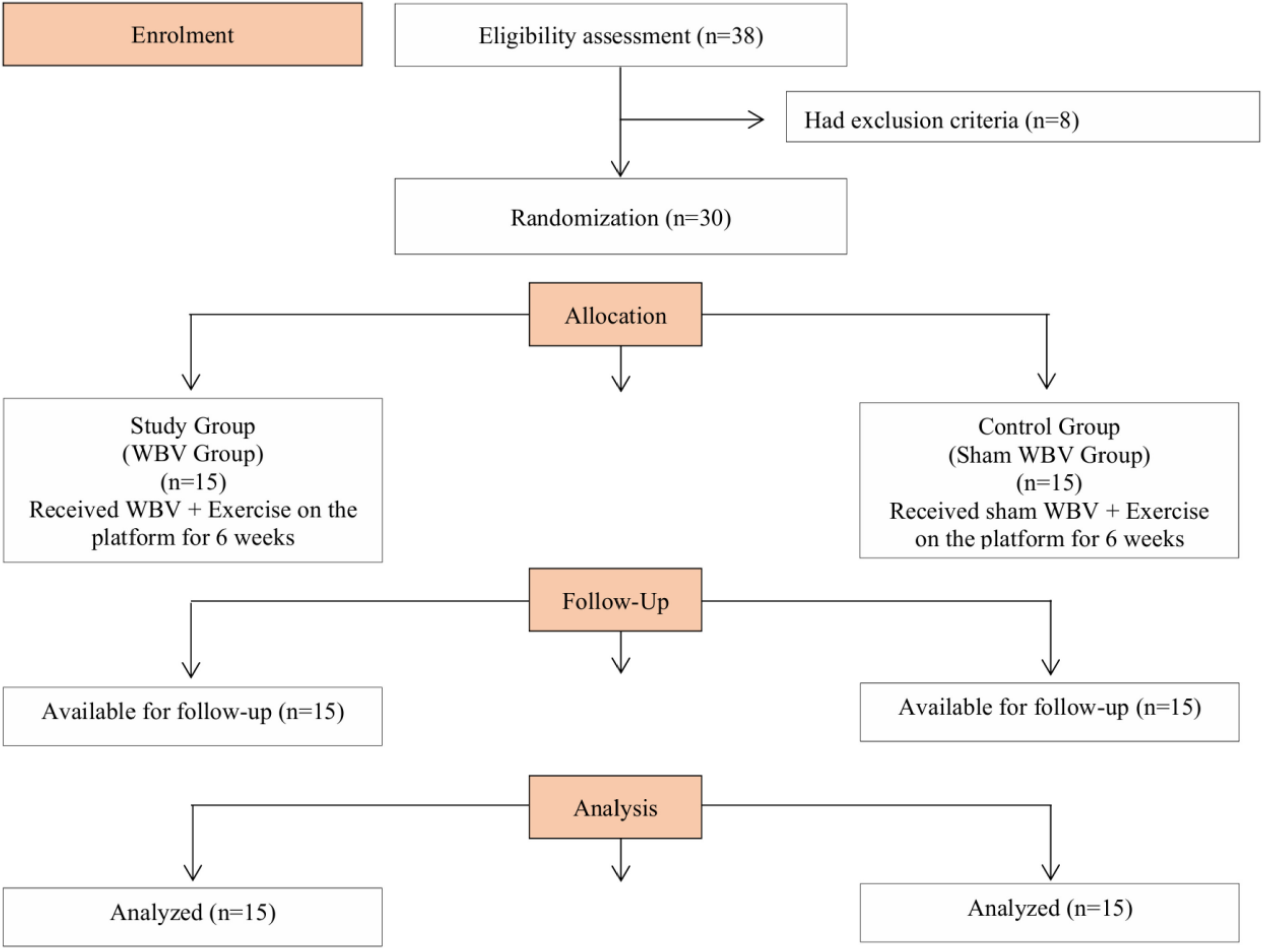
Participants’ flow chart throughout the trial.

To reduce potential bias in outcome assessment and interpretation, outcome assessors and data analysts were blinded to group allocation. Participants were informed that they would receive one of two vibration conditions but were not made aware of their specific group assignments. The intervention comprised either active whole-body vibration (WBV) training or sham vibration. In the sham condition, the platform did not produce any vibration; instead, a recorded vibration sound was played to simulate the auditory experience of the active intervention, thus maintaining participant blinding. No attrition was observed following randomization in either group (Figure 2).

### Procedures

Prior to the start of the study and providing of informed consent, all participants underwent an initial screening interview and comprehensive physical examination. These screening procedures were meticulously designed to identify any medical conditions that might contraindicate participation in whole-body vibration (WBV) training or potentially influence the study’s outcomes. Particular attention was given to detecting any previously diagnosed cardiorespiratory or neuromuscular disorders through a thorough review of the medical history and clinical assessment. Information regarding the current lifestyle and health behaviors of the participants was also collected.

### Intervention

Two groups of participants were formed: the experimental group (WBV group), comprising 15 individuals who underwent whole-body vibration (WBV) and selected exercises over a period of 6 weeks, and the control group (Sham WBV group), consisting of 15 individuals who received sham WBV alongside the same exercises for the same duration.

### Whole-body vibration

Each participant in the WBV group underwent WBV training for three weekly sessions, held on nonconsecutive days, for 6 weeks. WBV was performed using a Power Plate Pro 7HC (Northbrook, United States). The intensity of each session varied, and a 24-h break was required between sessions. Each 30-min session was structured with 15 min allocated to vibratory stimulation and 15 min to recovery, as evidence indicates that stimuli shorter than 20 min produce enhanced neuromuscular adaptations (32). The vibration parameters increased progressively over the intervention period. During the first week, a frequency of 25 Hz with 2 mm amplitude was applied, and the intensity gradually increased to 50 Hz with a 4 mm amplitude by the sixth week.

To facilitate the active contraction of the posterior leg muscles, specifically the soleus, lateral, and medial gastrocnemius muscles, three specific exercises were performed on a platform designed to enhance the ankle joint plantar flexion. The efficacy of this intervention was enhanced by incorporating muscle contraction with vibratory stimulation. In the initial exercise, participants positioned one leg in front of the other, with knees bent at an angle of approximately 110° to 120°, maintaining approximately one meter between the toes, which served as the support points for the feet. The second exercise required participants to squat on their toes, with their knees flexed at approximately 110° to 120°, and their feet spaced approximately 50 cm apart. The second and third exercises were similar; however, in the third exercise, the participant stood on one foot (33).

### Sham whole-body vibration

Participants assigned to the sham WBV group performed the same exercises as previously described, while standing on a deactivated WBV plate during three weekly sessions held on nonconsecutive days.

### Dietary control

Participants were advised to maintain their habitual dietary patterns during the study period. Dietary intake was not controlled or assessed in the study protocol.

### Outcome measures

#### Primary outcome measures

Anthropometric measurements, including Body Mass Index (BMI) and waist circumference (WC), were performed. BMI was calculated using the formula: weight (kg) divided by height (m^2^). An elevated BMI was classified as overweight (BMI ≥ 25) or obese (BMI ≥ 30). Height was determined as the average of two measurements obtained using a stadiometer or the closest two of three measurements if the discrepancy exceeded 0.5 cm. Weight was measured using a smart scale, based on the mean of two readings, or the nearest two out of three readings if the discrepancy was greater than 0.5 kg. WC was measured using a tape positioned at the midpoint between the inferior margin of the ribcage and the superior border of the iliac crest. The analysis utilized the average of two measurements, or three if the difference exceeded 0.5 cm (34).

#### Secondary outcome measures

Body composition analysis was performed using the Bioelectrical Impedance Analysis (BIA) method with a smart scale (eufy by Anker, Wi-Fi fitness tracking smart scale P3, Model No. 194644122041, China) according to the manufacturer’s guidelines. BIA is a widely recognized noninvasive technique for estimating body composition in both research and clinical settings. It has demonstrated high test–retest reliability and accuracy, proving useful for monitoring changes in body composition over time (35, 36). The secondary outcomes of interest in evaluating the efficacy of WBV in altering body composition included whole BF%, visceral fat, and lean body mass (kg) measurements. Body composition was assessed using electrical resistance, with personal information such as age, gender, weight, and height submitted to the manufacturer’s mobile application. The device was positioned on a level, hard surface according to the manufacturer’s instructions. The participants stood barefoot in a standing position, with their feet slightly apart, in the center of the scale until the measurement was completed, ensuring no movement and that their feet were dry. All measurements were conducted between 8 and 10 a.m., following an overnight fast of at least 8 h, as food or drink intake may influence BF% levels (37). Pre-treatment testing was conducted before the first session, and post-treatment testing occurred after 6 weeks of either real or sham WBV exposure. To enhance the measurement reliability, all assessments were performed under standardized conditions. Each measurement was repeated three times, and the average value was used for analysis.

### Statistical analysis

All statistical analyses were performed using the Statistical Package for the Social Sciences (SPSS) software version 23 for Windows. Box’s test for covariance homogeneity and the Shapiro-Wilk test for normality of data indicated no significant differences (*p* > 0.05). Consequently, a 2 × 2 mixed design Multivariate Analysis of Variance (MANOVA) was employed with an alpha level of 0.05 to examine variables across test groups and measurement timepoints.

## Results

### Demographic and subject characteristics

Age and sex distribution comparisons between the groups revealed no statistical significance (*p* > 0.05), as shown in Table 1.

**TABLE 1:** Demographics of participants in both groups.

Variables	WBV group	Sham WBV group	Comparison		Significance
	Mean ± SD	Mean ± SD	*t*-value	*P*-value	S
Age (years)	20.66 ± 2.05	20.6 ± 1.84	0.093	0.926	NS
**Sex distribution N (%)**	**WBV group**	**Sham WBV group**	**X^2^**	***P*-value**	**S**
Female	8 (53.4%)	9 (60%)	0.136	0.713	NS
Male	7 (46.6%)	6 (40%)			

SD: standard deviation; P: probability; S: significance; NS: non-significant.

### Effect of treatment on BMI, WC, BF%, visceral fat, and lean body mass

#### Multivariate analysis

A repeated-measures MANOVA demonstrated a significant effect of time on the combined dependent variables, namely BMI, WC, BF%, visceral fat, and lean mass, *F*(5, 24) = 158.08, *p* < 0.001, η^2^*p* = 0.971, indicating overall changes from pre- to post-intervention. Additionally, a significant time × group interaction was observed [*F*(5, 24) = 147.22, *p* < 0.001, η^2^*p* = 0.968], suggesting that temporal changes differed significantly between the WBV and control groups. However, the main effect of the group was not statistically significant [*F*(5, 24) = 1.86, *p* = 0.139, η^2^p = 0.280].

### Univariate analyses

#### Body mass index (BMI)

The analysis revealed a significant within-group effect of time [*F*(1, 28) = 556.95, *p* < 0.001, η^2^p = 0.952]. Additionally, a significant interaction between time and group was observed [*F*(1, 28) = 548.48, *p* < 0.001, η^2^p = 0.951].

#### Waist circumference (WC)

A significant effect of time was observed [*F*(1, 28) = 44.71, *p* < 0.001, η^2^p = 0.615], along with a significant time × group interaction [*F*(1, 28) = 21.71, *p* < 0.001, η^2^*p* = 0.437].

#### Body fat percentage (BF%)

There was a significant time effect, *F*(1, 28) = 10.18, *p* = 0.003, η^2^p = 0.267, and a significant time × group interaction, *F*(1, 28) = 25.18, *p* < 0.001, η^2^p = 0.473.

#### Visceral fat

A significant effect of time was observed [*F*(1, 28) = 63.18, *p* < 0.001, η^2^p = 0.693], with a significant time × group interaction [*F*(1, 28) = 50.58, p < 0.001, η^2^*p* = 0.644].

#### Lean mass

No significant time effect was found for the lean mass, *F*(1, 28) = 1.29, *p* = 0.265, η^2^p = 0.044, and no significant time × group interaction, *F*(1, 28) = 0.32, *p* = 0.574, η^2^p = 0.011.

#### Effect size interpretation

Effect sizes were determined and reported using partial eta squared (η^2^p) for all main and interaction effects obtained from the repeated-measures MANOVA. The magnitude of the effect sizes was interpreted according to conventional thresholds, where η^2^p = 0.01 indicated a small effect, η^2^p = 0.06 indicated a medium effect, and η^2^p ≥ 0.14 indicated a large effect. In the present study, the most significant outcomes demonstrated large effect sizes, indicating the strong practical significance of the intervention. Tables 2, 3 present the descriptive statistics and the intra- and inter-group differences with 95% confidence intervals for all dependent variables.

**TABLE 2:** Descriptive statistics and multiple pairwise comparison tests (*post-hoc* tests) for BMI and WC in the study and control groups at various time points.

MEASURE	WBV GROUP (MEAN ± SD)	SHAM WBV GROUP (MEAN ± SD)	95% CI	*P*-VALUE**
BMI (KG/M2)				
PRE-INTERVENTION	31.69 ± 4.29	31.88 ± 4.18	−3.367:2.98	0.902
POST INTERVENTION	28.21 ± 4.42	31.87 ± 4.20	−6.888: −0.432	0.028
95% CI	3.266:3.694	−0.201:0.228		
*P*-VALUE*	0.0001	0.9		
WAIST CIRCUMFERENCE (WC)				
PRE-INTERVENTION	108.43 ± 12.8	115.73 ± 12.04	−16.598:1.998	0.119
POST-INTERVENTION	100.6 ± 11.45	114.33 ± 11.65	−22.38: −5.087	0.003
95% CI	5.833:9.833	−0.6:3.4		
P-VALUE*	0.0001	0.163		

*p*-value *, within-group comparison; *p*-value **, between-group comparison, 95% CI, 95% confidence interval.

**TABLE 3:** Descriptive statistics and multiple pairwise comparison tests (*post-hoc* tests) for BF%, visceral fat, and lean mass variables for the study and control groups at various measurement times.

MEASURE	WBV GROUP (MEAN ± SD)	SHAM WBV GROUP (MEAN ± SD)	95% CI	*P*-VALUE**
BODY FAT %				
PRE-INTERVENTION	41.9 ± 4.79	39.92 ± 5.14	−1.744:5.691	0.286
POST-INTERVENTION	40.07 ± 4.83	40.33 ± 4.84	−3.88:3.36	0.884
95% CI	1.182:2.471	−1.051:0.238		
*P*-VALUE*	0.0001	0.207		
VISCERAL FAT				
PRE-INTERVENTION	9.2 ± 2.59	9 ± 2.39	−1.667:2.067	0.828
POST-INTERVENTION	8 ± 2.32	8.93 ± 2.40	−2.704:0.837	0.29
95% CI	0.969:1.431	−0.164:0.297		
*P*-VALUE*	0.0001	0.559		
LEAN BODY MASS (KG)				
PRE-INTERVENTION	48.96 ± 10.06	53.67 ± 9.46	−12.015:2.602	0.198
POST-INTERVENTION	48.94 ± 10.03	53.61 ± 9.35	−11.926:2.592	0.199
95% CI	−0.082:0.122	−0.042:0.162		
*P*-VALUE*	0.691	0.238		

*p*-value *, within-group comparison; *p*-value **, between-group comparison, 95% CI, 95% confidence interval.

#### Within-group comparisons

**FIGURE 3:**
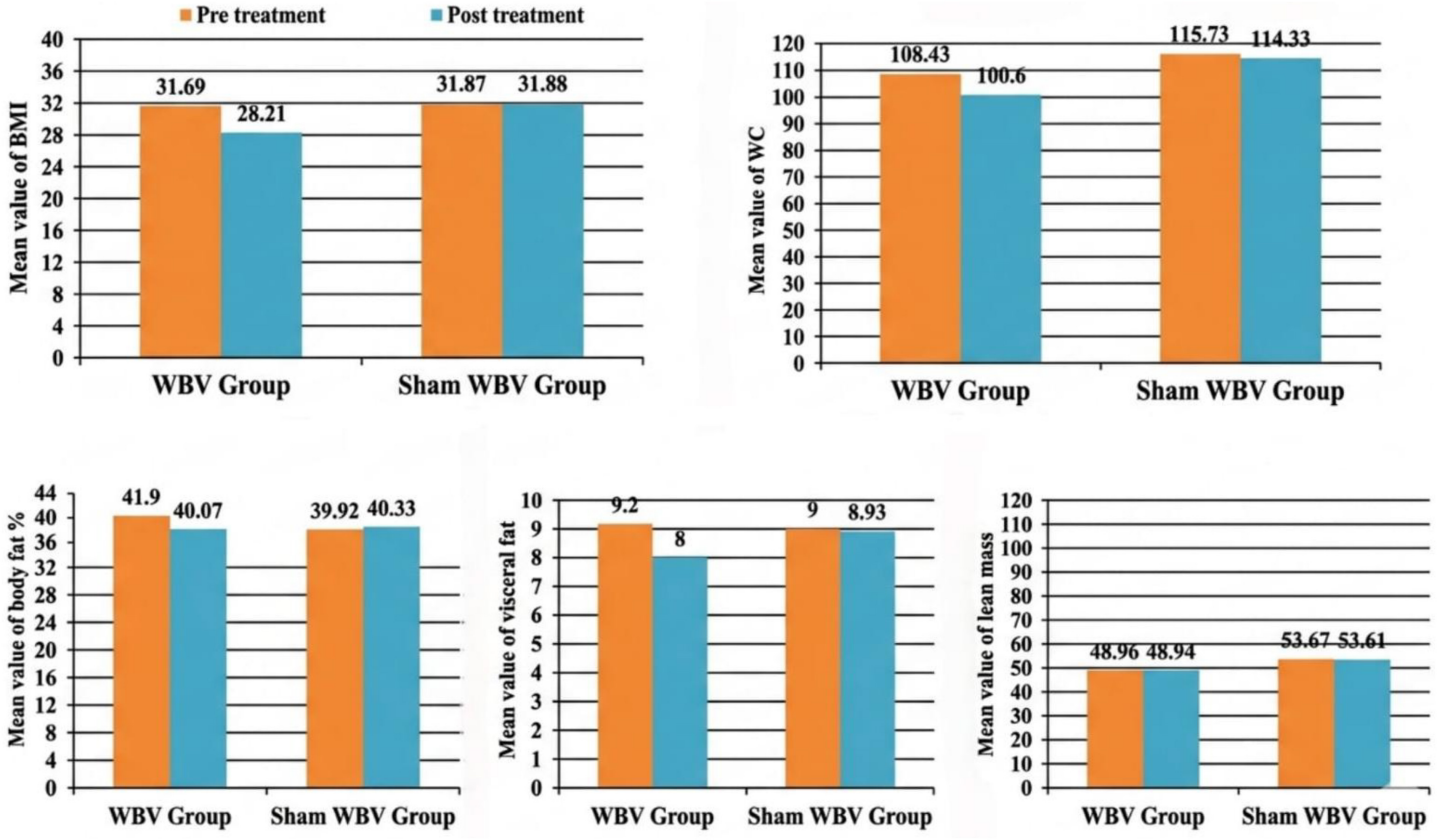
Mean values of BMI, WC, BF%, visceral fat and lean mass pre- and post-intervention in both groups.

Within-group pairwise comparisons demonstrated that the WBV group exhibited statistically significant reductions (*p* < 0.05) in BMI, waist circumference (WC), BF%, and visceral fat after the intervention, whereas lean mass remained unchanged (*p* > 0.05) relative to baseline values. In contrast, the sham WBV group showed no statistically significant changes (*p* > 0.05) in any of the assessed outcomes (Figure 3).

#### Between-group comparisons

Between-group comparisons indicated no significant differences in BF%, visceral fat, or lean mass at either time point. However, BMI and WC showed significantly greater reductions (*p* < 0.05) in the WBV group than in the sham group post-intervention (Figure 3).

#### Adverse effects

During the trial, the participants were actively supervised and monitored for any adverse effects during and after each intervention session. No significant side effects were observed; only one participant experienced mild leg paraesthesia that resolved immediately after the session.

## Discussion

This randomized controlled trial demonstrated that the WBV intervention yielded significant improvements over time, particularly in anthropometric measurements such as BMI and waist circumference. Although significant interaction effects were observed for several variables, the between-group differences at specific time points were confined to BMI and waist circumference, while other body composition variables did not exhibit statistically significant differences.

Following treatment, the WBV group’s BMI and WC significantly decreased (*p* < 0.05) compared to the sham WBV group. These results are partially consistent with those of Iconaru et al. (37), who demonstrated that a WBV exposure program effectively enhanced participants’ lipid metabolism and nutritional health. The treatment plan resulted in a reduction in weight and BMI (37). Song et al. (38) also investigated WBV exercise, and concluded that it might aid in lowering WC and body weight in women after menopause. However, postmenopausal healthy obese women should also take care to maintain their muscle mass by including appropriate resistance training and nutrition (38). It is postulated that in women over age 65, the catabolic effects of vibration on adipose tissue may be attributed to the conversion of kinetic energy into thermal energy through frictional forces (39). In addition, mouse studies suggest that exposure to vibration mimics certain metabolic effects typically associated with physical exercise (40).

In the current study, no significant differences were observed in BF%, visceral fat, or lean mass between groups, indicating that the intervention did not produce measurable changes in body composition in this cohort of young adults. This finding is consistent with previous WBV studies reporting minimal or no effects on fat mass and lean mass (41–45). Importantly, similar outcomes have been observed in both young individuals and postmenopausal women, despite differences in hormonal status and metabolic characteristics, suggesting that WBV alone may provide an insufficient anabolic or lipolytic stimulus. The absence of change may also be attributed to intervention duration and the lack of combined strategies such as resistance training or nutritional modification. From a clinical perspective, these results suggest that WBV should not be considered an effective standalone intervention for improving body composition, but rather as an adjunct to more established approaches targeting fat reduction and muscle mass enhancement.

In contrast to the current study, which showed no significant changes in BF%, visceral fat, or lean mass, previous studies have reported beneficial effects of WBV on body composition. For instance, Alavinia et al. (21) suggested that WBV can significantly reduce fat mass, particularly when combined with dietary and exercise interventions (21), while Vissers et al. (30) reported greater reductions in visceral adipose tissue in obese adults following WBV compared to aerobic exercise (30). Multimodal approaches may be necessary to achieve meaningful changes. Notably, WBV-induced improvements in body composition have been more consistently observed when applied as part of structured weight management programs, where reductions in visceral fat and total fat mass are more likely to occur (46–48). However, findings across the literature remain inconsistent, and discrepancies may be attributed to differences in the studied population, intervention duration, training protocols, and whether WBV was used as a standalone treatment or combined with other weight loss strategies.

It is important to note that a substantial proportion of the literature examining WBV effects on body composition has been conducted in postmenopausal or older women, whose hormonal profiles, baseline body composition, and metabolic responses differ markedly from those of the young adult population included in the present study. These differences, particularly in estrogen status, resting metabolic rate, and fat distribution, may influence the magnitude and nature of the response to WBV. Therefore, while such studies provide valuable mechanistic insights, their findings should be interpreted with caution when applied to college-aged individuals. The current results contribute to addressing this gap by specifically evaluating WBV in a younger cohort, highlighting that the intervention may have more limited effects on body composition when used in isolation in this population.

## Strengths and limitations

This current study had several notable strengths. First, it evaluated a range of anthropometric and body composition measurements, including BMI, WC, BF%, visceral fat, and lean mass, within a single experimental design. Second, the inclusion of a sham control group contributed to strengthening the methodological rigor of the study and allowed for a more reliable assessment of the effects of WBV training.

Despite these findings, several limitations must be acknowledged when interpreting our results. The relatively small sample size (*n* = 30) may have limited statistical power and reduced the ability to detect subtle effects. Additionally, recruitment from a single university population may constrain the external validity and generalizability of the outcomes. The relatively short intervention duration of 6 weeks, coupled with the absence of follow-up assessments, precluded the evaluation of the sustainability of the observed effects over time. Moreover, the lack of control over dietary intake and habitual physical activity represents a potential source of confounding factors that may have influenced body composition outcomes.

Additionally, body composition was assessed using bioelectrical impedance analysis, which, although less precise than gold-standard techniques such as dual-energy X-ray absorptiometry (DEXA), is widely recognized in the literature as a valid, practical, and clinically sufficient method for estimating body composition in both research and clinical settings. Evidence indicates that BIA provides acceptable accuracy for group-level comparisons and population-based studies, particularly when standardized measurement conditions are applied (49, 50).

Finally, despite efforts to resemble the auditory experience of the intervention in the sham group, complete participant blinding may not have been achieved because of the inherent nature of the WBV mechanical vibration stimulus.

## Conclusion

WBV positively impacts anthropometric parameters in overweight and obese college students by enhancing BMI and WC, potentially making it a significant component of forthcoming weight-loss initiatives. However, WBV did not have a significant effect on body composition, which may warrant further investigation in future studies. A distinct body of literature highlights the limitations and variability in the effectiveness of whole-body vibration (WBV) in modifying body composition. Recognizing these discrepancies is crucial for developing effective interventions aimed at addressing obesity among young adults. Consequently, future research with larger sample sizes, extended durations, adequate follow-up, and improved control of dietary intake and physical activity will be highly beneficial.

## Data Availability

The original contributions presented in this study are included in this article/supplementary material, further inquiries can be directed to the corresponding author.
